# Diaphragmatic Ultrasonography in Sports Performance: A Systematic Review

**DOI:** 10.3390/life14101250

**Published:** 2024-10-01

**Authors:** Harold Andrés Payán-Salcedo, Florencio Arias-Coronel, Jose Luis Estela-Zape, Maria Fernanda Serna-Orozco

**Affiliations:** 1 Faculty of Health, Universidad Santiago de Cali, Cali 760024, Valle del Cauca, Colombia; 2 Research Group Salud y Movimiento, Universidad Santiago de Cali, Cali 760024, Valle del Cauca, Colombia

**Keywords:** sports performance, ultrasonography, diaphragm

## Abstract

This paper aims to investigate and analyze the correlation between diaphragmatic parameters through ultrasonography and sports performance in various sports disciplines. This systematic review followed the PRISMA methodology. The search strategy was applied in the Medline database through Ovid, EMBASE, LILACS, the Cochrane Central Register of Controlled Trials, and Open Gray. Clinical trials, cohort, case–control, and cross-sectional studies were included, and animal experiments were excluded. A total of 388 studies were identified. After removing duplicates and screening titles and abstracts, sixteen studies were selected for full review, and six were included in the qualitative analysis. The results demonstrated a positive correlation between diaphragm excursion and thickness during inspiration with the anaerobic power, highlighting their importance in high-intensity performance. Additionally, one study reported a positive correlation between diaphragm thickness and aerobic power, suggesting the need for further research. The impact of inspiratory muscle training in Paralympic athletes was also evaluated, providing valuable insights into diaphragmatic adaptation in disabled populations. Ultrasonography is a feasible tool for evaluating the structure and function of the diaphragm, the main element of the respiratory process during sports practice. Its use could contribute to the evaluation and planning of sports training and be a possible indicator of performance improvement.

## 1. Introduction

Sports performance hinges on the intricate interplay between the biomechanical and physiological system [1], with the diaphragm and respiratory functions playing a pivotal role [2]. As the primary respiratory muscle, the diaphragm is vital for pulmonary ventilation during physical activity, ensuring efficient gas exchange and oxygen supply to tissues necessary for energy production [3,4]. Additionally, the diaphragm aids in trunk stabilization and strength generation, working in tandem with other muscles to maintain the integrity of athletic movements [5,6].

The strength of the diaphragm is crucial for various sports activities, including running, swimming, and weightlifting [7,8]. The connection between diaphragmatic strength and athletic performance is profound, as it contributes to trunk stability, effective breathing, postural support, and increased intra-abdominal pressure. These factors facilitate the transfer of force from the trunk to the extremities, thereby enhancing movements, jumps, strokes, or throws. Conversely, diaphragmatic dysfunction can adversely affect respiratory efficiency, trunk stability, and force generation during physical activity [9,10].

Research [11,12,13] has demonstrated that deficiencies in diaphragmatic strength can impair athletic performance and elevate the risk of injury. Such deficiencies compromise respiratory capacity, induce muscle tension and fatigue, and restrict trunk stability and respiratory efficiency. This combination of factors leads to decreased performance and a heightened risk of muscle injury [14].

Ultrasound has proven to be a valuable tool for emphasizing the importance of respiratory training in sports and for monitoring specific movements through noninvasive techniques. Integrating ultrasound into training programs enables coaches and athletes to directly observe diaphragmatic activity, detect dysfunctions, and make necessary adjustments to training protocols. This approach enhances the precision of monitoring and intervention, thereby optimizing performance and preventing injuries [15].

A diaphragmatic ultrasound is employed to evaluate muscle thickness and respiratory function, as well as to monitor specific adaptations resulting from training [16]. In healthy adults, the reference values for diaphragmatic thickness range from approximately 2.2 to 2.8 mm. These values may vary based on factors such as age, sex, and individual physiological differences. Typically, higher diaphragmatic thickness is observed in younger individuals and athletes, reflecting more robust muscle development. Conversely, diaphragm thickness generally decreases with age, and gender differences are noted, with women exhibiting slightly lower values compared to men due to anatomical and physiological variations [12,15,16].

However, the accuracy of diaphragmatic ultrasound results depends on the adherence to a standardized protocol, which includes measurements taken in the supine or semi-recumbent position. B-mode imaging assesses diaphragmatic excursion and structural integrity, while M-mode imaging measures diaphragmatic thickness and contractile activity during respiration [15]. Ensuring precision requires operator training and multiple measurements due to the diaphragm’s inherent variability. Comparisons with reference values from different populations—considering factors such as age, sex, and physical activity levels—should be avoided to prevent inaccuracies [13]. These practices are essential for accurate assessment of diaphragmatic function and its effects on respiratory capacity and athletic performance [17].

Ultrasonography measures the thickness of the diaphragm, evaluates movement during breathing, and detects potential dysfunctions such as reduced mobility, injury, or muscle atrophy [18,19]. Integrating this tool into sports training programs facilitates the development of personalized approaches to improve performance and mitigate the risks associated with diaphragmatic dysfunction [20]. Although the relationship between diaphragmatic ultrasound findings and sports performance has yet to be systematically investigated, there is a clear need for a comprehensive review to analyze and understand this correlation across different sporting disciplines.

## 2. Materials and Methods

This study was conducted in accordance with the recommendations of the Cochrane Collaboration and the PRISMA statement. The protocol was registered in the Prospective International Register of Systematic Reviews (PROSPERO) CRD42024503708.

### 2.1. Eligibility Criteria

The studies were included if they evaluated sports performance in adult or child athletes from various disciplines, including Paralympic sports, and assessed diaphragmatic parameters through ultrasonography, correlating these with performance. Additionally, secondary outcomes included studies that reported measurements of diaphragmatic and abdominal muscle functions measured through peak respiratory, inspiratory, and expiratory pressures. There were no time limits.

### 2.2. Source of Information

A search strategy was devised for Medline (OVID), Scopus, Web Of Science, LILACS, and the Cochrane Central Register of Controlled Trials (CENTRAL). The search strategy was specific to each database and included a combination of medical titles and free-text terms for ultrasonography, sports performance, and study type. A targeted search was conducted using indexed terms and free writing from sources of conference abstracts, ongoing clinical trials (accessed in November 2023), literature published in non-indexed journals, and other gray literature sources. A generic search strategy was designed for Google Scholar. No language restrictions or restrictions on the publication status of the article were considered. Articles published from inception up to December 2023 were included. The full search strategy for each database can be found in Appendix A.

### 2.3. Data Collection

Two investigators independently and blindly reviewed the titles and abstracts of articles within the systematic review to determine their potential usefulness. Eligibility criteria were applied during the full-text review of potentially eligible articles. The two researchers resolved discrepancies in consensus. In cases without consensus, a third reviewer made the final decision. Data were collected by two investigators using a standardized extraction tool with the following information for each article: study design, geographical location, authors’ names, title, objectives, inclusion and exclusion criteria, number of patients included, tests and measures, outcome definitions, results and measures of association, and source of funding.

### 2.4. Methodological Quality Assessment

The Newcastle–Ottawa scale (for case–control and cross-sectional studies) was used for methodological quality assessment of the studies, as recommended by the Cochrane Collaboration. The Newcastle–Ottawa scale contains eight items, classified into three dimensions with a score of 0–9 points, and the studies were classified as low quality (0–2 points), fair quality (3–5 points), and good/high quality (6–9 points) [21]. Clinical trials were assessed using the PEDro scale, which examines the randomization process, blinding, and outcome processing. A score on the PEDro scale above 6/10 was considered “good” [22]. Discrepancies between authors during the quality assessment were resolved through discussion. If no agreement was reached, a third author was consulted.

## 3. Results

### 3.1. Study Selection

**Figure 1:**
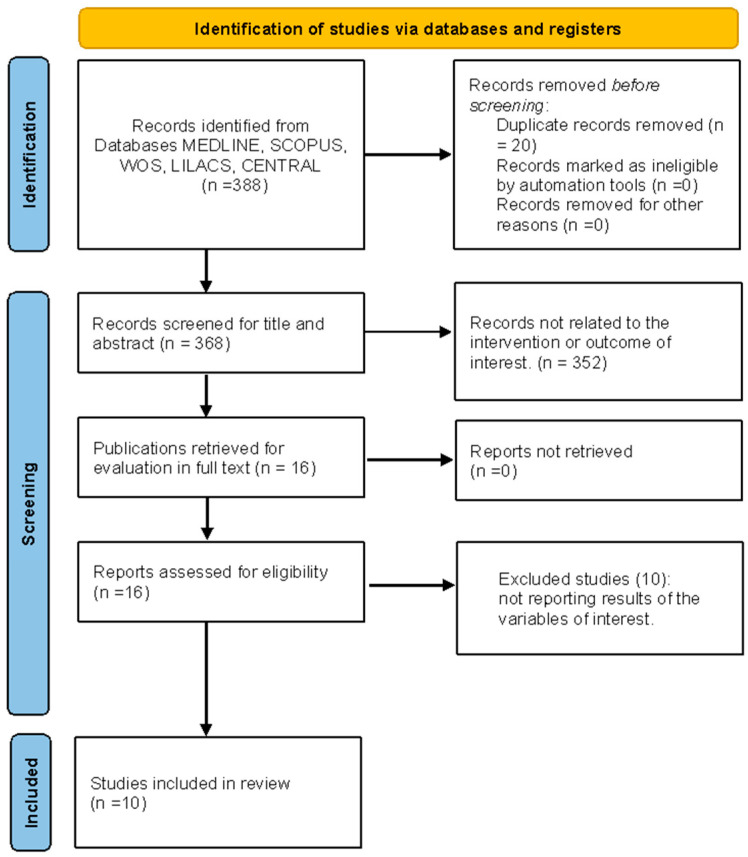
Flowchart of study selection.

A total of 388 studies were identified from the search. After excluding duplicates, 368 studies were reviewed, and 16 were selected for full-text review. Ten of these were excluded because they were medical treatment protocols, leaving six studies for inclusion in this review: Farias et al., 2023 [23], Ichiba et al., 2020 [24], Palac et al., 2023 [25], Erail et al., 2022 [26], West et al., 2013 [27], and Brown et al., 2013 [28] (Figure 1).

### 3.2. Characteristics of Excluded Studies

Articles not related to the intervention or outcome of interest were excluded. Additionally, letters to the editor, systematic reviews, narrative reviews, drug intervention protocols, and animal studies were excluded.

### 3.3. Characteristics of Included Studies

The six studies included in this review were published between 2013 and 2023. Three of the studies included exclusively male athletes [25,26,28], while the others included both male and female athletes [23,24,27]. The sample sizes ranged from 10 to 63 participants. Two of the articles focused on athletes from Paralympic sports—boccia [24] and wheelchair rugby [27]—while the other studies included weightlifting athletes [28], football players [25], and multi-sport athletes [23,26] (Table 1).

Five of the included studies [23,24,25,26,28] aimed to determine the relationship between lung function, diaphragmatic characteristics, and performance in sport-specific tests. One study focused on assessing improvements in respiratory structure and function in response to inspiratory muscle training [27]. The latter study was the only controlled clinical trial included in the review and the only study evaluating the effects of an inspiratory muscle training program (Table 1).

### 3.4. Risk of Bias and Methodological Quality Assessment

The results of the methodological assessment of the observational studies are presented. Three of the five cross-sectional studies were classified as having good methodological quality, while two were classified as having fair quality. Most studies exhibited a high risk of bias due to the lack of blinded assessment and the inability to pre-estimate the sample size. The evaluation of clinical trials is shown in Table S2. In the context of clinical trials, no risk of bias was found, and the assessed study was classified as having a high methodological quality.

### 3.5. Results of the Association between Diaphragmatic Thickness/Excursion and Sports Performance

Five studies [23,25,26,27,28] assessed diaphragmatic thickness, and two studies [24,25] analyzed diaphragmatic excursion using ultrasonography. Among the studies measuring diaphragmatic thickness, three [26,27,28] reported significantly higher values in athletes compared to control groups. The relationship between diaphragmatic thickness and oxygen consumption showed variability: two studies [23,25] found no significant correlation; one study [26] identified a significant negative correlation, and another study [27], which employed an inspiratory muscle training protocol, observed a non-significant trend toward increased diaphragmatic thickness and oxygen consumption in the training group relative to the control group. Additionally, studies evaluating cycle ergometer work rate [26,27] found higher work rates in athlete groups compared to control and placebo groups, revealing a positive relationship between diaphragmatic thickness and work rate during testing (Table 2).

Regarding sport-specific assessments, three studies [24,25,28] reported significant findings. Brown et al. (2013) documented a positive correlation between the total weight lifted in competition and diaphragmatic thickness. Ichiba et al. (2020) identified a significant correlation between diaphragmatic displacement, body weight, and throwing distance in boccia athletes. Conversely, Palac et al. (2023) found a non-significant negative correlation between diaphragmatic thickness and running test results in soccer players, although they observed a significant positive correlation between diaphragmatic excursion and performance in the running test [24,25] (Table 2).

These discrepancies suggest that both diaphragmatic thickness and excursion are influenced by the intensity and type of physical training. The absence of a significant correlation between diaphragmatic thickness and oxygen consumption in some studies may indicate that oxygen consumption, which reflects aerobic capacity and cardiovascular efficiency, does not always correlate directly with diaphragmatic thickness. Additionally, variations in results could be attributed to the role of other respiratory muscles, such as the intercostals and abdominals, which also contribute to respiratory function and may affect diaphragmatic observations depending on the sport and training regimen applied.

### 3.6. Results of the Association between Peak Respiratory Pressures and Sports Performance

Four studies included measurements of peak inspiratory pressures (PImax) and peak expiratory pressures (PEmax) [24,26,27,28]. Two of the studies [26,28] reported higher levels of both PImax and PEmax in the athlete groups compared to the control groups. Brown et al. (2013) found a positive correlation between PImax, PEmax, and diaphragmatic thickness in athletes compared to untrained controls, with no significant differences in lung function between the two groups. Conversely, West et al. (2013) reported no correlation between diaphragmatic thickness and inspiratory strength following an inspiratory muscle training protocol in athletes with spinal cord injuries. Ichiba et al. (2020) were the only authors to report a correlation between PImax, PEmax, and physical performance tests (throwing distance) in boccia athletes, finding a positive correlation between both variables (Table 2).

## 4. Discussion

This review indicates that diaphragm thickness during inspiration is positively correlated with anaerobic power [24,25,26], suggesting that increased thickness may enhance performance in high-intensity activities. Conversely, only one study [26] reported a positive correlation between diaphragm thickness and aerobic power, implying that this relationship is less pronounced. Aerobic power appears to be more closely associated with cardiovascular efficiency and oxygen transport rather than diaphragmatic thickness. Additionally, a study [27] on inspiratory muscle training in Paralympic athletes demonstrated that while such training can alter diaphragm morphology, it does not lead to significant improvements in aerobic power. The lack of a significant correlation between aerobic power and respiratory parameters may be due to the fact that endurance sports, while beneficial for cardiovascular health, do not produce the same morphological changes in the diaphragm as anaerobic sports. Respiratory training has been shown to enhance respiratory muscle strength and endurance [29,30,31,32], which may account for the observed variations in diaphragm morphology and function among athletes.

Adult athletes engaged in individual sports, such as judo and wrestling, demonstrate increased diaphragmatic thickness compared to athletes participating in team sports, such as football, basketball, and volleyball. Research by Holtzhausen et al. [33] indicates that both individual and team sport athletes exhibit higher diaphragmatic thickness values compared to sedentary individuals. However, the precise mechanisms underlying these differences between sport types remain inadequately defined. It is hypothesized that training specific to team sports, which emphasizes endurance and predominantly relies on aerobic energy systems, may lead to a reduction in muscle fiber size and the cross-sectional area of respiratory muscles [34]. This adaptation is thought to enhance oxygen utilization efficiency, potentially resulting in a relatively smaller diaphragmatic thickness in these athletes [35]. Conversely, the increased diaphragmatic thickness observed in athletes of individual sports may represent a physiological adaptation to the anaerobic demands of such activities, aimed at optimizing muscle function for high-intensity performance, rather than signifying pathological hypertrophy.

Much lower values of diaphragmatic thickness were reported in adolescent football players compared to adult athletes. This can be attributed to the maturation process of muscle metabolism and the progressive, asynchronous transition to an adult metabolic profile, which involves changes in body size and composition, increases in the cross-sectional areas of type I and II fibers [36], and gradual improvements in muscle strength and a more balanced oxidative and glycolytic enzyme activity from adolescence to adulthood. These physiological changes influence athletic performance over time [37].

In strength sports such as powerlifting, studies [28] have shown that these athletes possess higher levels of diaphragmatic thickness and maximal inspiratory pressure (PImax) compared to non-athletes. This can be explained by the adaptations in musculoskeletal physiology induced by the discipline, including an increase in the cross-sectional area of type II fibers and a conversion of type IIX fibers to IIA [38,39,40]. These findings align with other research, which has documented significant increases in PImax and PEmax in strength-trained athletes compared to those trained for endurance [17].

One clinical trial included in this review established that a 6-week inspiratory muscle training protocol for wheelchair rugby athletes resulted in significant increases in diaphragmatic thickness, PEmax, and VO2max. While similar outcomes have been documented for various sports [41,42,43,44,45], most studies have focused on the impact of this training on lung function, ventilation, and sports performance without incorporating structural assessments of the diaphragm through ultrasonography.

In two studies included in this research, West et al. [27] and Brown et al. [28] presented important discrepancies in their results regarding the correlation between diaphragmatic thickness and sports performance variables. These, we consider, can be explained by the marked difference between the study population (powerlifters and athletes with spinal cord injury), training regimens, and differences in the baseline parameters of lung function and respiratory muscle strength.

The review encompassed a wide range of sports, including both individual and team disciplines. Notably, two studies involved high-performance athletes with spinal cord injuries competing in wheelchair rugby and boccia. These studies demonstrated that this population has lower respiratory muscle strength and diaphragmatic excursion compared to predicted values. As demonstrated by West et al. [27] in their placebo-controlled experimental study of 10 Paralympic wheelchair rugby players with complete spinal cord injury, those who received pressure threshold IMT for 6 weeks, with 30 dynamic inspiratory maneuvers, twice daily, 5 days a week, showed significant changes in diaphragm thickness compared to the placebo group, suggesting it as a strategy for increasing muscle strength and improving sports performance; although due to the small sample size (n = 10), these results should not be generalized. It is interesting to note that, in their study, the placebo group presented a slight decrease in the values of diaphragmatic thickness, PImax, and PEmax after 6 weeks of intervention; this further evidences and highlights the importance of IMT on diaphragm hypertrophy and increased respiratory muscle strength in this population. Supporting these findings, other research observed significant increases in inspiratory and expiratory pressures, as well as VO_2_max and exercise capacity, in wheelchair rugby athletes following a 6-week respiratory muscle training protocol [46]. The physiological mechanisms underlying the increase in inspiratory muscle strength in this population are not fully understood. However, researchers such as Hardy et al.; Ref. [47] suggest that inspiratory muscle training may improve hemodynamics and exercise capacity by enhancing respiratory muscle pump function.

Most of the studies included in this review were characterized by the evaluation of the diaphragmatic dome excursion (DoD) using a low-frequency transducer in an abdominal window and diaphragmatic thickening using a high-frequency linear transducer in the zone of apposition (ZOA); these two methods are conventional and widely used; however, their results between operators may suffer from high variability and affect their interpretation. Due to this, a systematic approach for the evaluation of diaphragmatic thickness and excursion [48] has been proposed, which offers greater simplicity and potentially more reproducible results, independent of the hepatic and splenic acoustic windows. This approach, which uses a 38 mm high-frequency (10–15 MHz) linear probe in the coronal plane, above the midaxillary line, and evaluates the distance traveled by the most cranial portion of the zone of apposition (ZOA) from the end of expiration to the end of inspiration during breathing at vital capacity, has already been validated and found to have a higher inter-rater reliability [49] than the two conventional approaches.

It is well known that diaphragmatic ultrasound is used in a variety of settings and contexts. In intensive rehabilitation, diaphragmatic ultrasound is essential for predicting the success of weaning from mechanical ventilation [50]. Studies [51,52,53] have demonstrated that diaphragmatic excursion and thickness are reliable indicators, with cut-off values of 11–14 mm for excursion and a thickening fraction of 30–36%. These measurements enable precise adjustments to respiratory treatment protocols, promoting an effective transition to spontaneous breathing.

In post-ICU patients, who frequently exhibit diaphragmatic weakness due to immobility and prolonged mechanical ventilation, diaphragmatic ultrasound provides an accurate evaluation of diaphragmatic function and structure. This facilitates the monitoring of respiratory capacity and informs targeted strengthening interventions. Exercises developed to enhance diaphragmatic function in athletes can be adapted for post-ICU patients, helping to strengthen the weakened diaphragm and improve respiratory volumes and capacities [54,55,56] Consequently, diaphragmatic ultrasound is critical in both weaning from mechanical ventilation and post-ICU rehabilitation and may support the transition to sports-oriented rehabilitation programs.

The data from this review underscore the potential of diaphragmatic ultrasonography as a valuable tool in sports. Although its use in sports is currently limited, the results highlight its importance for comprehensive athlete evaluation and the development of specific training strategies aimed at enhancing performance. The measurements of diaphragmatic thickness and excursion are indicative of the muscle’s structure and function, and inefficient use of this muscle can be a limiting factor in performance.

Despite the comprehensive review and detailed data analysis, significant limitations exist. The scarcity of the included studies and the diversity of study designs and sample sizes, measurement methods, and participant characteristics made pooled analyses and the interpretation of results difficult, hindering the ability to draw definitive conclusions. Additionally, including athletes with motor limitations and from various sports adds complexity to result interpretation.

Further research, particularly clinical trials, is recommended to explore diaphragmatic function measures in sports performance to deepen our understanding in this area.

## 5. Conclusions

A diaphragmatic ultrasound is a promising tool that should be implemented in sports to optimize training programs and assess muscle adaptations, as it provides valuable data on diaphragmatic structure and function. Although increases in diaphragmatic thickness have been observed, its functional relevance to sports performance remains unclear. The use of a diaphragmatic ultrasound complements but does not replace respiratory function testing, although it could improve the personalization of training and the assessment of athletes’ respiratory health. However, further research and standardized protocols are needed to validate its clinical effectiveness.

## Figures and Tables

**Table 1:** Characteristics of included studies.

Scheme	Type of Study	Objective	n Total	Sex (%M)	Type of Sports	Age * (n)	Main Findings
Brown et al., 2013 (UK) [28]	Control-Case	To investigate the functional changes in the ventilatory system of elite powerlifters	20	50	Powerlifting	Powerlifting (10): 28 ± 11.3/untrained (10): 25 ± 4.1	Maximal pulmonary pressures and diaphragm thickness were greater in powerlifters compared to controls.
West et al., 2013 (ENG) [27]	Randomized controlled trial	To determinate if inspiratory muscle training (IMT) improves respiratory structure and function and peak exercise responses in highly trained athletes with cervical spinal cord injury (SCI).	10	90	Wheelchair rugby	Placebo group (5): 27.9 ± 2.8/IMT group (5): 30.5 ± 2.2	IMT resulted in significant diaphragmatic hypertrophy and increased inspiratory muscle strength in highly trained athletes with cervical SCI.
Farias et al., 2023 (BR) [23]	Cross-sectional	Assess the association between diaphragm thickness and the physical performance of athletes and the effects of COVID-19 infection on these parameters.	63	81	Soccer referee, karate, cycling, soccer, athletics, swimming, running, triathlon, martial arts, and judo	Males (51): 23.44 ± 9.65; Females (12): 16.67 ± 5.03	No significant association between diagram thickness and maximum oxygen consumption.
Ichiba et al., 2020 (JP) [24]	Cross-sectional	Determine the relationship between pulmonary function, pitching distance, and psychologicalcompetitive ability of Japanese boccia players.	13	77	Boccia	32.9 ± 12.0	Significant correlations between diaphragm excursion, weight, and pitching distance.
Erail et al., 2022 (TR) [26]	Case–control	Examine the correlation between the aerobic and anaerobic performance of diaphragm thickness in athletes	40	100	Soccer, basketball, volleyball players, individual athletes, sprinter, wrestling, and judo	TA (15): 21.80 ± 2.40 IA (15): 18.93 ± 2.31 CON (10): 23.60 ± 2.91	Greater diaphragmatic thickness in athletes correlates with anaerobic activities and thinner diaphragm with higher VO2Max levels.
Palac et al., 2023 (POL) [25]	Cross-sectional	Determine the relationship between ultrasound imaging of respiratory muscles during tidal breathing and running tests	22	100	Football players	17.1 ± 0.29	Diaphragmatic excursion is positively correlated with 5 m and 10 m speed tests, respectively, in adolescent football players.

* Age is expressed in mean ± SD; n: size of subgroup sample; TA: team athletes; IA: individual athletes; CON: control group; IMT: inspiratory muscle training; SCI: spinal cord injury.

**Table 2:** Results of included studies.

Scheme	Measure	Subgroups	Variable	Results	*p* Value	Correlation
Brown et al., 2013 [28]	Respiratory muscle function	Powerlifter	Diaphragm thickness in expiration (mm)	3.10 ± 0.99	<0.01	NR
		Untrained		2.06 ± 0.33		
		Powerlifter	Maximal static inspiratory pressure (cmH_2_O)	−156.8 ± 24.9	<0.05	r = 0.518; *p* = 0.019 ^a^
		Untrained		−127.7 ± 35.5		
		Powerlifter	Maximal static expiratory pressure (cmH_2_O)	199.9 ± 66.4	0.07	r = 0.671; *p* = 0.001 ^a^
		Untrained		153.4 ± 41.6		
	Sports Performance	Powerlifter	Muscle strength test (kg)	735 ± 211.3	<0.05	r = 0.825; *p* = 0.03 ^a^
		Untrained		no measured		
West et al., 2013 [27]	Respiratory muscle function	Placebo	Diaphragm thickness (mm)	Pre: 3.42 ± 0.21/Post: 3.32 ± 0.20	0.001	NR
		IMT		Pre: 3.13 ± 0.17/Post: 3.79 ± 0.06		
		Placebo	Maximum static inspiratory pressure (cmH_2_O)	Pre: −122 ± 12/Post: −116 ± 15	0.017	r = 0.25, *p* = 0.67 ^a^
		IMT		Pre: −121 ± 17/Post: −135 ± 15		
		Placebo	Maximum static expiratory pressure (cmH_2_O)	Pre: 72 ± 16/Post: 71 ± 15	<0.01	NR
		IMT		Pre: 78 ± 19/Post: 94 ± 19		
	Sports Performance	Placebo	Work rate (W)	Pre: 53.3 ± 13.7/Post: 54.3 ± 15.3	0.034	NR
		IMT		Pre: 54.9 ± 5.9/Post: 63.1 ± 6.1		
		Placebo	Oxygen uptake-VO_2_ (L/min)	Pre: 1.11 ± 0.24/Post: 1.08 ± 0.28	0.077	NR
		IMT		Pre: 1.08 ± 0.17/Post: 1.27 ± 0.12		
Farias et al., 2023 [23]	Respiratory muscle function	All participants	Diaphragm thickness (%)	Females: 61.00 ± 0.2	0.22	NR
				Males: 55.00 ± 0.25		
	Sports performance		Maximal oxygen uptake-VO_2_ Max (mL/kg/min)	Females: 39.34 ± 1.74		r = 0.30; *p* = 0.22
				Males: 41.25 ± 6.84		
Ichiba et al., 2020 [24]	Respiratory muscle function	All participants	Diaphragm excursion (mm)	Resting inspiration: 1.76 ± 0.45	NR	r = 0.598, *p* = 0.05 ^b^
				Resting expiration: 1.42 ± 0.47	NR	r = 0.620, *p* = 0.05 ^b^
				Maximal inspiration: 3.9 ± 1.36	NR	r = 0.589, *p* = 0.05 ^b^
				Maximal expiration: 1.05 ± 0.31	NR	NR
			Maximum static inspiratory pressure (cm/H_2_O)	−53.1 ± 25.4	NR	NR
			Maximum static expiratory pressure (cm/H_2_O)	50.7 ± 25.1	NR	
	Sport performance		Pitching distance (mts)	18.09 ± 7.86	NR	
Erail et al., 2022 [26]	Respiratory muscle function	IA	Maximum static inspiratory pressure (cmH_2_O)	−129.80 ± 28.93	0.010 *	r = −0.010 ^d^; r = 0.283 ^c^; *p* > 0.05
		TA		−110.67 ± 24.99	NR	r = −0.122 ^d^; r = −0.295 ^c^; *p* > 0.05
		CON		−94.8 ± 26.36	NR	r = −0.211 ^d^; r = 0.082 ^c^; *p* > 0.05
		IA	Maximum static expiratory pressure (cmH_2_O)	168.87 ± 48.73	0.001 *	r = 0.156 ^d^; r = 0.064 ^c^; *p* > 0.05
		TA		134.67 ± 26.07	NR	r = −0.009 ^d^; r = 0.333 ^c^; *p* > 0.05
		CON		102.6 ± 29.31	NR	r = −0.269 ^d^; r = −0.011 ^c^; *p* > 0.05
		IA	Diaphragmatic thickness in inspiration (mm)	5.67 ± 1.19	0.048	Reported correlations with other parameters with letter ^c^
		TA		4.87 ± 1.16		
		CON		4.56 ± 1.19		
		IA	Diaphragmatic thickness in expiration (mm)	1.94 ± 0.43	0.001	Reported correlations with other parameters with letter ^d^
		TA		1.61 ± 0.21		
		CON		1.40 ± 0.25		
	Sport performance	IA	Average power (W)	619.99 ± 127.74	0.008 *	r = 0.483 ^c^; *p* < 0.05
		TA		556.37 ± 96.05	NR	r = 0.085 ^d^; r = 0.306 ^c^; *p* > 0.05
		CON		469.71 ± 93.94	NR	r = 0.054 ^d^; r = 0.180 ^c;^ *p* > 0.05
		IA	Peak power (W)	878.33 ± 207.59	0.008 *	r = 0.495 ^c^; *p* < 0.05
		TA		749.47 ± 137.41	NR	r = 0.477 ^c^; *p* < 0.05
		CON		649.25 ± 159.08	NR	r = −0.04; r = −0.049; *p* > 0.05
		IA	Maximal oxygen uptake-VO_2_ Max (mL/kg/min)	54.39 ± 7.07	0.006 *	r = −0.551 ^c^; *p* < 0.05
		TA		57.56 ± 6.93	0.001 *	r = 0.169 ^d^; r = -0.230 ^c^; *p* > 0.05
		CON		46.76 ± 3.68	NR	r = −0.095 ^d^; r = 0.049 ^c^; *p* > 0.05
Palac et al., 2023 [25]	Respiratory muscle function	All participants	Diaphragm excursion (cm)	4.73 ± 1.45	NR	r = 0.46; *p* = 0.04 ^e^
			Diaphragm thickness end of tidal inspiration (mm)	2.09 ± 0.85	NR	Reported correlations with other parameters with letter ^c^
			Diaphragm thickness end of tidal expiration (mm)	1.71 ± 0.59	NR	Reported correlations with other parameters with letter ^d^
	Sport performance		Shuttle run test (total number of completed 20 m repetitions)	127 ± 13.2	NR	r = 0.01, *p* = 0.98 ^c^; r = 0.10, *p* = 0.66 ^d^
			Maximal oxygen uptake-VO_2_ Max (mL/kg/min)	56.2 ± 3.54	NR	r = 0.17, *p* = 0.45 ^c^; r = 0.29, *p* = 0.18 ^d^
			Speed test-distance 5 m (s)	1.03 ± 0.005	NR	r = −0.07, *p* = 0.75 ^c^ r = −0.27, *p* = 0,23 ^d^
			Speed test-distance 10 m (s)	1.87 ± 0.52	NR	r = −0.06, *p* = 0.8 ^c^; r = −0.12, *p* = 0.6 ^d^
			Speed test-distance 30 m (s)	4.19 ± 0.20	NR	r = 0,22, *p* = 0.34 ^c^; r = 0.25, *p* = 0.25 ^d^

NR: not reported; TA: team athletes; IA: individual athletes; CON: control group; IMT: inspiratory muscle training; SCI: spinal cord injury; ^a^ correlated with diaphragm thickness; ^b^ correlated with pitching distance; ^c^ correlated with diaphragm thickness in inspiration; ^d^ correlated with diaphragm thickness in expiration; * compared to controls; ^e^ correlated with speed test results.

## Data Availability

The raw data supporting the conclusions of this article will be made available by the authors on request.

## Supplementary Materials

The following supporting information can be downloaded at https://www.mdpi.com/article/10.3390/life14101250/s1, Table S1. Methodological assessment of included studies with New Castle Ottawa scale; Table S2. Methodological assessment of included studies with New Castle Ottawa scale; Table S3. Methodological assessment of included studies with PEDro scale.

## Appendix A

Search Strategy:

Medline (Ovid)

Athletes.mp AND (diaphragm Ultrasonography).mp or (diaphragm thickness).mp or (diaphragm excursion).mp or (respiratory muscles).mp AND (Athletic Performance or exercise testing).mp AND randomized controlled trial.pt or controlled clinical trial.pt or randomized.ab or placebo.ab or randomly.ab or trial.ab or (clinical adj2 trial).mp or (randomi*ed adj2 controlled adj2 trial).mp or exp double-blind method or Non-Randomized Controlled Trials as Topic or (non*randomized adj2 trial*).mp or exp cohort studies or (cohort* adj2 stud*).mp or exp case–control studies or (case*control adj2 stud*).mp or exp cross-sectional studies or (cross*sectional adj2 stud*).mp.

Scopus

(TITLE-ABS-KEY (athlete*) AND TITLE-ABS-KEY (“diaphragm Ultrasonography” OR “diaphragm thickness” OR “diaphragm excursion” OR “respiratory muscles”) AND ALL (“Athletic Performance” OR “exercise testing”) AND ALL (“randomized controlled trial” OR “controlled clinical trial” OR randomized OR placebo OR randomly OR trial OR “clinical NEXT2 trial” OR “randomi*ed NEXT2 trial” AND “controlled NEXT2 trial” OR “double-blind method” OR “non-randomized” OR “controlled trials” OR “non*randomized NEXT2 trial * “ OR “cohort studies” OR “cohort* NEXT2 stud*” OR “case-control studies” OR “case*control NEXT2 stud*” OR “cross-sectional studies” OR “cross*sectional AND NEXT2 stud*”)).

Web of Science

((AB = (athlete*)) AND AB = ((“diaphragm Ultrasonography” OR “diaphragm thickness” OR “diaphragm excursion” OR “respiratory muscles”)) AND ALL = ((“Athletic Performance” OR “exercise testing”)) AND ALL = ((“randomized controlled trial” OR “controlled clinical trial” OR randomized OR placebo OR randomly OR trial OR “clinical NEXT2 trial” OR “randomi*ed NEXT2 trial” AND “controlled NEXT2 trial” OR “double-blind method” OR “non-randomized” OR “controlled trials” OR “non*randomized NEXT2 trial* “ OR “cohort studies” OR “cohort* NEXT2 stud*” OR “case-control studies” OR “case*control NEXT2 stud*” OR “cross-sectional studies” OR “cross*sectional AND NEXT2 stud*”)).

Central (Ovid)

Athletes.mp AND (diaphragm Ultrasonography).mp or (diaphragm thickness).mp or (diaphragm excursion).mp or (respiratory muscles).mp AND (Athletic Performance or exercise testing).mp AND randomized controlled trial.pt or controlled clinical trial.pt or randomized.ab or placebo.ab or randomly.ab or trial.ab or (clinical adj2 trial).mp or (randomi*ed adj2 controlled adj2 trial).mp or exp double-blind method or Non-Randomized Controlled Trials as Topic or (non*randomized adj2 trial*).mp or exp cohort studies or (cohort* adj2 stud*).mp or exp case–control studies or (case*control adj2 stud*).mp or exp cross-sectional studies or (cross*sectional adj2 stud*).mp.

Lilacs

mh: (atleta* OR deportista*) AND tw: (“ultrasonografia” OR “grosor diafragmatico” OR “excursion diafragmatica” OR “musculos respiratorios”) AND tw: (“rendimiento deportivo” OR “rendimiento”) AND tw: (ensayo clinico OR doble ciego OR experimento clínico OR casos controles OR transversal OR cohorte).

Google Scholar

Ultrasonography OR diaphragm OR diaphragm thickness OR respiratory muscles AND Athletes AND Athletic Performance OR exercise testing.
