# Supplementary material for: Diaphragmatic Ultrasonography in Sports Performance: A Systematic Review
Source: Life (Basel). 2024 Oct 1;14(10):1250. doi: 10.3390/life14101250 (PMC11508651; doi:10.3390/life14101250)
Supplement: Supplementary file 1 [file life-14-01250-s001.zip › life-3141860-supplementary.pdf]

Table S1. Methodological assessment of included studies with New Castle Ottawa scale—*Case control studies*

**First author & reference**	**Selection**				**Comparability**	**Exposure**			**Score**
	Is the Case Definition Adequate?	Representativeness of the Cases	Selection of Controls	Definition of Controls	Comparability of cases and controls	Ascertainment of exposure	Same method of ascertainment for cases and controls	Non-Response rate	Total
Brown et al., 2013	*	-	*	*	*	-	*	*	6/9
Erail et al., 2022	*	*	*	*	*	-	*	*	7/9

Table S2. Methodological assessment of included studies with New Castle Ottawa scale—*Cross sectional studies*

**First author & reference**	**Selection**				**Comparability**	**Exposure**			**Score**
	Is the eligibility criteria specified?	Representativeness of the sample	Selection of sample/sample size	Definition of no included subjects	Comparability between participants	Assessment of outcomes	Same method of Assessment of outcome for all the sample	Statistical test	Total
Farias et al., 2023	*	*	-	*	*	-	*	*	6/9
Palac et al., 2023	*	*	*	*	*	-	*	*	5/9
Ichiba et al., 2020	*	*	-	*	-	-	*	-	4/9

Table S3. Methodological assessment of included studies with PEDro scale

MAJOR COMPONENTS	West et al., 2013
1. Eligibility criteria were specified	1
2. Subjects were randomly allocated to groups (in a crossover study, subjects were randomly allocated an order in which treatments were received)	1
3. Allocation was concealed	1
4. The groups were similar at baseline regarding the most important prognostic indicators	1
5. There was blinding of all subjects	1
6. There was blinding of all therapists who administered the therapy	1
7. There was blinding of all assessors who measured at least one key outcome	1
8. Measures of at least one key outcome were obtained from more than 85% of the subjects initially allocated to groups	1
9. All subjects for whom outcome measures were available received the treatment or control condition as allocated or, where this was not the case, data for at least one key outcome was analyzed by "intention to treat"	1
10. The results of between-group statistical comparisons are reported for at least one key outcome	1
11. The study provides both point measures and measures of variability for at least one key outcome	1
